# 

**Published:** 1894-05

**Authors:** 


### CONTENTS—MAY, 1894.—VOL. IX., NO. 11.

ORIGINAL COMMUNICATIONS—

The Railway Surgeons and the Law. Clark Bell, Esq., New York . . 553

Leprosy. Isadore Dyer, M. D., New Orleans. . . . . . . . . . . . . . 558

Law vs. Justice in the Practice of Medicine and Surgery in Texas. Jas. Kennedy, M. D., Ph. G., Galveston . . . . . . . . . . . . . 565

ABSTRACTS OF CURRENT MEDICAL LITERATURE—

Department of Therapeutics. Edited by Prof. D. Cerna, M. D., Galveston. . . . . . . . . . . . . . . . . . . . . . . . . . 570

SOCIETY NOTES—

Texas State Medical Association—Twenty-sixth Annual Meeting . . 571

State Pharmaceutical Association . . . . . . . . . . . . . . . . 592

EDITORIAL—

Medical Department University of Texas . . . . . . . . . . . . . 593

A Libel on the Profession . . . . . . . . . . . . . . . . . . . 596

Commencement Day . . . . . . . . . . . . . . . . . . . . . . . . 600

That Banquet . . . . . . . . . . . . . . . . . . . . . . . . . . 602

A Merited Honor . . . . . . . . . . . . . . . . . . . . . . . . 604

Biographical—President-elect J. W. McLaughlin, M. D . . . . . . . 605

MEDICAL NEWS AND MISCELLANY—

Many Interesting Paragraphs . . . . . . . . . . . . . . . . . . 606

PUBLISHERS' NOTES . . . . . . . . . . . . . . . . . . . . . . . . 607

